# Prognostic Significance of the Lymphocyte-to-Monocyte Ratio in Bladder Cancer Undergoing Radical Cystectomy: A Meta-Analysis of 5638 Individuals

**DOI:** 10.1155/2019/7593560

**Published:** 2019-04-04

**Authors:** Jian-ying Ma, Gang Hu, Qin Liu

**Affiliations:** Department of Breast Surgery, Thyroid Surgery, Huangshi Central Hospital of Edong Healthcare Group, Hubei Polytechnic University, No. 141, Tianjin Road, Huangshi, Hubei, China

## Abstract

**Introduction:**

A growing number of studies have explored the association between the pretreatment lymphocyte-to-monocyte ratio (LMR) and survival outcomes in various cancers. However, its prognostic significance on bladder cancer remains inconsistent. The aim of this meta-analysis was to evaluate the prognostic value of pretreatment LMR in bladder cancer.

**Methods:**

The MEDLINE, EMBASE, Cochrane Library, and CNKI databases were comprehensively searched for relevant studies. A meta-analysis of overall survival (OS), recurrence-free survival (RFS), or cancer-specific survival (CSS) clinicopathological features was conducted.

**Results:**

Nine studies containing 5,638 cancer patients were analyzed in this meta-analysis. Patients with high LMR tended to have favourable OS (HR: 0.63, 95% CI: 0.50-0.80, *P* < 0.001), RFS (HR: 0.59, 95% CI: 0.38-0.91, *P* = 0.017), and CSS (HR: 0.76, 95% CI: 0.70-0.83, *P* < 0.001). Moreover, low LMR was highly correlated with age (≥60), differentiation (low), T stage (III-IV), lymph node metastasis (yes), and concomitant Cis (yes).

**Conclusion:**

Pretreatment LMR might be a useful predictor of survival outcomes in patients with bladder cancer.

## 1. Introduction

Bladder cancer is one of the most common urinary malignancies and represents approximately 90%-95% of urothelial carcinomas [1]. There was an estimated 76,960 newly diagnosed cases (58,950 men and 18,010 women) in the USA in 2016 [2]. Although the accuracy of current diagnostic methods has greatly improved, the 5-year overall survival (OS) remains unsatisfying, especially for metastatic bladder cancer [1, 2]. Furthermore, bladder cancer has a high recurrence rate (50%), and 15-40% of cases develop into a muscle-invasive form of the disease [3, 4]. Therefore, a reliable and readily accessible preoperative prognostic biomarker is required to determine the optimal therapeutic strategies.

A growing number of studies have shown that inflammation has been closely involved in tumorigenesis and cancer progression and also has been found to correlate with the prognosis [5]. Based on the accumulating evidence, inflammation-based models, such as the C-reactive protein/albumin ratio, albumin-to-globulin ratio (AGR), inflammation-based index (IBI), and neutrophil-to-lymphocyte ratio (NLR), and the platelet-to-lymphocyte ratio (PLR) have been developed to predict oncological outcomes in a variety of human cancers [6–9]. The lymphocyte-to-monocyte ratio (LMR) has been associated with worse prognoses in various cancers such as hepatocellular carcinoma, colorectal cancer, and lung adenocarcinoma [10–12]. However, because of the inconsistent results, whether LMR is associated with the prognosis in the bladder remains controversial [13–15]. Therefore, this study was conducted to evaluate the prognostic value of LMR and analyze the relationships between LMR and clinicopathological parameters in patients with bladder cancer.

## 2. Materials and Methods

### 2.1. Search Strategies

The MEDLINE, EMBASE, Cochrane Library, and CNKI databases were comprehensively searched up to September 20th, 2018. Studies focused on the correlation of LMR and bladder cancer were taken into retrieved. Studies were selected using the following keywords: “bladder cancer,” “bladder carcinoma,” “bladder adenocarcinoma,” “bladder tumor,” “bladder neoplasms,” “transitional cell carcinoma,” “ureteral neoplasms,” or “urethral neoplasms” and “LMR,” “lymphocyte to monocyte ratio,” “lymphocyte monocyte ratio,” or “lymphocyte-to-monocyte ratio.” The references of retrieved studies were also checked to avoid missing relevant studies. For the detailed search strategies, please refer to the supplementary material file (available here).

### 2.2. Selection Criteria

The criteria for inclusion were as follows: (1) studies focused on bladder cancer patients, (2) studies that evaluated the prognostic value of LMR, (3) a reported cut-off value for LMR, and (3) available hazard ratios (HRs) with 95% CIs for OS, RFS, or CSS. Articles were excluded if they were duplicate publications or reviews, data was not usable, or the studies were only performed on animals.

### 2.3. Data Extraction and Quality Assessment

Data extraction and quality evaluation were independently operated by two investigators. Any discrepancies between the two investigators were resolved by discussion until reaching a consensus. General information was gathered as follows: the first authors' name, year of publication, age, geographical regions, number of patients, distribution of gender, sample size, tumor stage, differentiation, lymph node metastasis, distant metastasis, treatment type, tumor size, cut-off values, survival outcome, follow-up period, and concomitant Cis (carcinoma in situ).

The methodological quality of included studies was independently assessed according to the Newcastle-Ottawa Scale (NOS) [16], which included three primary domains: Selection, Comparability, and Outcome. Studies with an NOS score of ≥6 were deemed high-quality studies.

### 2.4. Statistical Analysis

We used Stata 13.0 statistical software (Stata, College Station) to estimate HRs for OS, RFS, and CSS and odds ratios (ORs) for clinicopathological parameters. As for prognostic variables (e.g., OS, DFS, and RFS), the hazard ratio (HR) and corresponding 95% confidence interval (CI) were directly extracted from published studies. Otherwise, they were indirectly calculated from survival curves if only survival curves were available in some studies [17, 18]. The heterogeneity among studies was assessed using Cochran's *Q* test and Higgins *I* ^2^ statistic; the fixed effect model was used in case of the absence of significant heterogeneity (*P* > 0.10 or/and *I* ^2^ < 50%); otherwise, the random effect model was chosen. We then performed subgroup analyses to examine the potential source of heterogeneity. To validate the robustness of the pooled results, sensitivity analyses were performed by removing each study. Publication bias was evaluated using Egger's test. All *P* values were two-sided, and the difference was considered significant when the *P* value was less than 0.05.

## 3. Results

### 3.1. Study Characteristics

**Figure 1:**
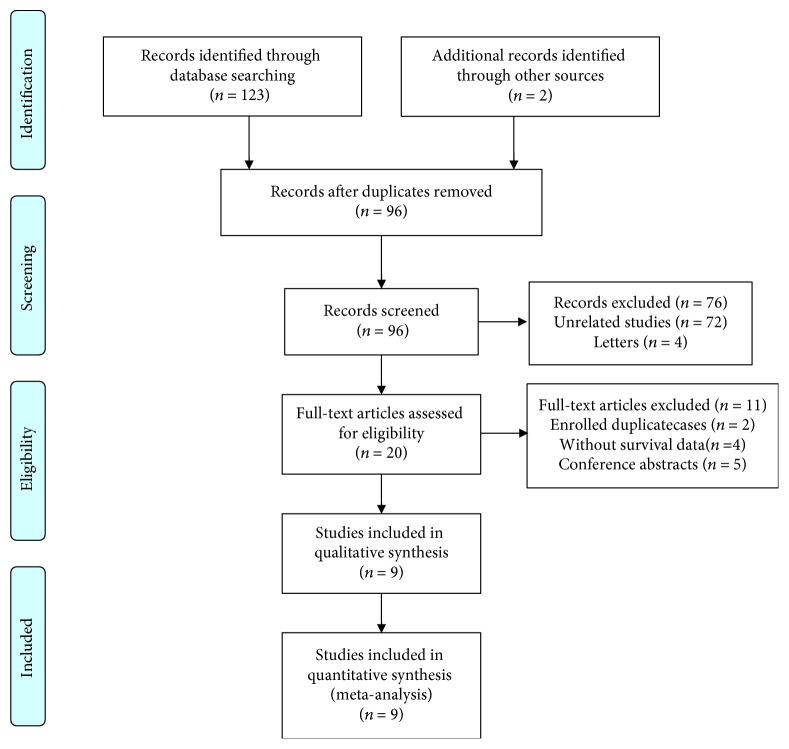
The flow chart of the study selection procedure in the meta-analysis.

A total of 123 records were initially retrieved from four common databases (Figure 1). Meanwhile, 2 articles were identified through references. After the removal of duplicates, 96 articles remained for further evaluation. As shown in Figure 1, after screening titles and abstracts, 76 duplicate articles were excluded. The remaining articles were carefully evaluated by evaluating the full texts, and 11 articles were excluded. Finally, a total of 9 studies were included in the meta-analysis [13–15, 19–24].

Table 1 displays the characteristics of included studies. All included studies were retrospective cohort trials and released from years 2014 to 2018. These studies were carried out in six countries, including Lebanon, UK, China, Japan, Austria, and Poland. The treatments were surgery and mixed methods. Cut-off values of LMR ranged from 1.8 to 4. All cases in the eligible studies were classified into two groups (high and low). Eight studies reported the association between LMR and OS, and 3 studies reported RFS and CSS. NOS scores of all the studies were at least 6 or more (Table 1).

## 4. Meta-Analysis

### 4.1. Impact of LMR on OS

**Figure 2:**
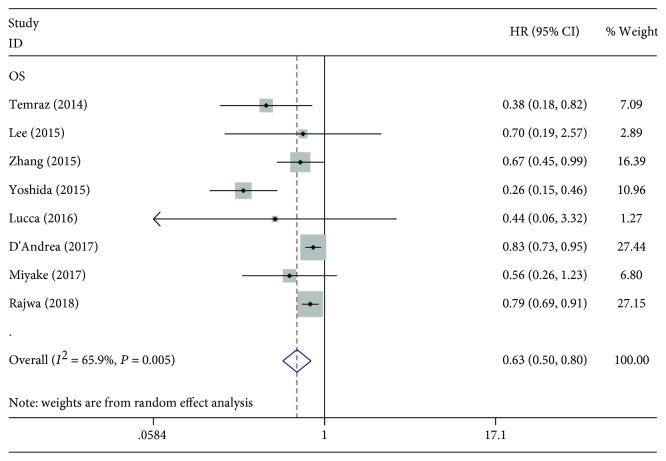
Forest plot of the correlation between LMR and OS in bladder cancer patients.

Eight studies, including 5,368 patients, were included in this meta-analysis of OS. The pooled results showed significant differences in OS among the higher LMR groups and lower LMR groups (HR: 0.63, 95% CI: 0.50-0.80, *P* < 0.001, Figure 2). To further explore the prognostic value of LMR in bladder cancer, subgroup analysis based on the main features was performed. Results for subgroup analyses are shown in Table 2. The results indicated that elevated LMR significantly predicted favourable OS in patient mixed stages (HR = 0.63; 95% CI = 0.49-0.80; *P* < 0.001). Pooled HRs for OS were stratified by the cut-off value for LMR. The result showed that patients with LMR ≥ 3 had significantly increased OS (HR: 0.56, 95% CI: 0.35-0.88, *P* = 0.011). However, no prognostic value was observed in patients with LMR < 3 (HR: 0.65, 95% CI: 0.41-1.04, *P* = 0.075). Moreover, the ethnicity, treatment, and analysis method also did not affect the significant predictive value of LMR in bladder cancer patients.

### 4.2. Impact of LMR on RFS and CSS

**Figure 3:**
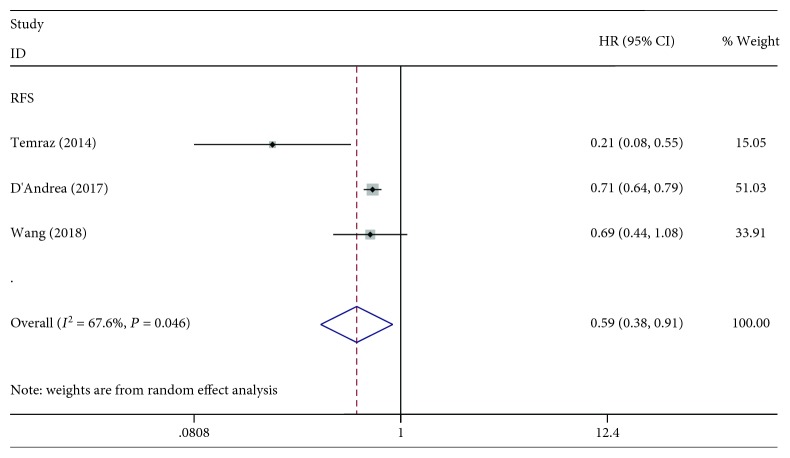
Forest plot of the correlation between LMR and RFS in bladder cancer patients.

**Figure 4:**
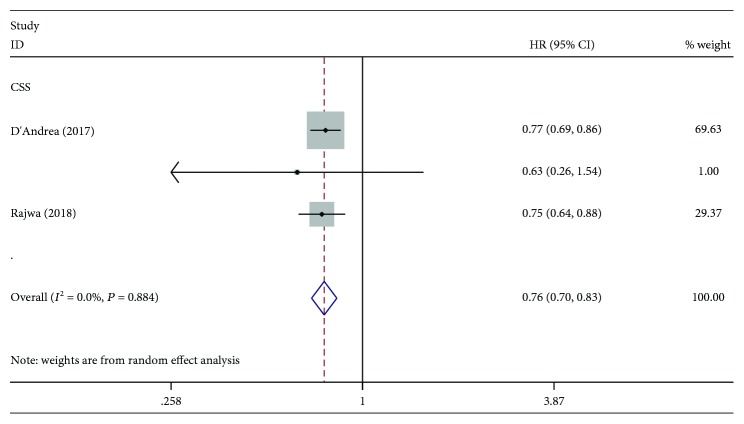
Forest plot of the correlation between LMR and CSS in bladder cancer patients.

Three studies with 4,536 patients investigated the association between LMR and RFS. The pooled HR was 0.59, which indicated that elevated LMR was significantly associated with favourable RFS (Figure 3). There were three studies with a total of 4,459 patients investigating the predictive value of LMR for CSS. As shown in Figure 4, a statistically significant difference was observed between the higher LMR groups and the lower LMR groups (HR: 0.76, 95% CI: 0.70-0.83, *P* < 0.001). The result revealed that patients with a high LMR had a significantly favourable CSS compared with those with a low LMR.

### 4.3. Associations between LMR and Clinicopathological Parameters

Meta-analyses for the association between LMR and clinicopathological parameters were conducted, and the results are presented in Table 3. Compared with high LMR, low LMR was highly correlated with age (≥60 vs. <60; OR = 2.07, 95% CI: 1.22-3.50, *P* = 0.007), differentiation (low vs. moderate/high; OR = 1.60, 95% CI: 1.10-2.32, *P* = 0.01), T stage (III-IV vs. I-II; OR = 1.13, 95% CI: 1.01-1.28, *P* = 0.04), lymph node metastasis (yes vs. no; OR = 1.22, 95% CI: 1.06-1.39, *P* = 0.005), and concomitant Cis (yes vs. no; OR = 0.88, 95% CI: 0.78-0.99, *P* = 0.03). However, there was no obvious relationship between the LMR and gender (male vs. female; OR = 1.18, 95% CI: 0.68-2.04, *P* = 0.56), smoking status (always/current vs. never; OR = 0.95, 95% CI: 0.63-1.45, *P* = 0.82), tumor size (>3 cm vs. <3 cm; OR = 1.86, 95% CI: 0.74, 4.71, *P* = 0.19), distant metastasis (yes vs. no; OR = 1.46, 95% CI: 0.37-5.73, *P* = 0.59), and multiplicity (multiple vs. solitary; OR = 1.04, 95% CI: 0.68-1.58, *P* = 0.86).

### 4.4. Sensitivity Analysis and Publication Bias

**Figure 5:**
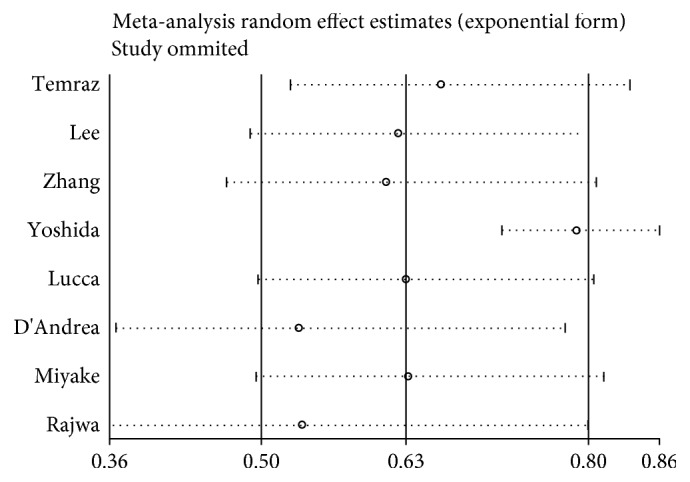
Sensitivity analysis of OS for LMR.

A sensitivity analysis was performed by omitting the enrolled studies in turn to investigate the stability of the results. The results indicated that the pooled results were relatively reliable and steady (Figure 5).

**Figure 6:**
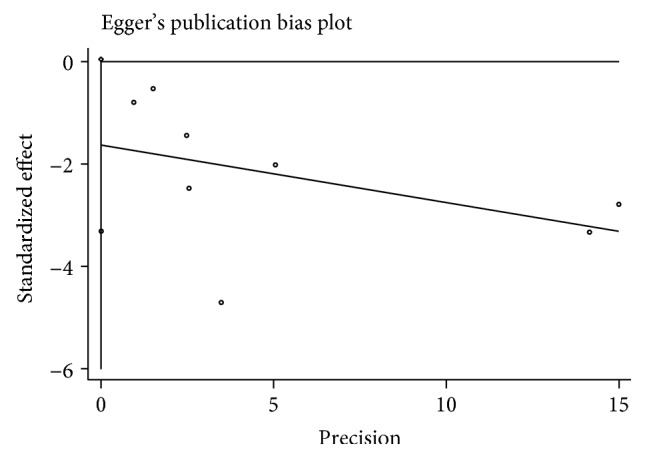
Egger's publication bias plot of OS in bladder cancer.

No obvious publication bias was found among studies (Figure 6), which were also demonstrated in Egger's test for OS (*P* > ∣*t*∣ = 0.055).

## 5. Discussion

Recently, more and more studies focused on the correlation between inflammation and cancers revealed that tumor initiation, progression, and metastasis were affected by host systemic inflammatory response as well as tumor microenvironment [5, 25, 26]. To the best of our knowledge, our study is the first and most comprehensive meta-analysis that systematically analyzed the prognostic value of pretreatment LMR in bladder cancer survivors. A prognostic effect for LMR on OS, RFS, and CSS was found after pooling the results. Therefore, LMR could serve as biomarker for the prognosis of bladder cancer patients. Additionally, the correlations between LMR and clinicopathological parameters were evaluated. Low LMR was highly correlated with age (≥60), differentiation (low), T stage (III-IV), lymph node metastasis (yes), and concomitant Cis (yes).

LMR, as a composite inflammatory-based prognostic system, has shown great prognostic value in multiple cancers. However, the underlying molecular mechanisms have not been adequately illuminated. Lymphocytes play a major role in suppressing cancer cell proliferation and migration [27]. Tumor-infiltrating lymphocytes (TILs) are vital components of the antitumor immune microenvironment and are involved in several stages of tumor progression [28, 29]. Cytotoxic lymphocytes, mainly cytotoxic T cells, are essential for eliminating residual cancer cells and are being applied in immunotherapy [30, 31]. Monocytes are thought to have an impact on tumorigenesis through differentiation to tumor-associated macrophages (TAMs). TAMs are recruited to the tumor site by obtaining the signal from tumor-derived chemotactic factors [32]. Therefore, the amount and percentage of monocytes could be representative for TAMs reflecting the tumor burden. Recent studies reported that increased infiltration of TAMs was associated with the outcome of various cancers [33, 34]. Thus, LMR may represent a balance between the antitumor immune reaction and the tumor promotion function.

There were several limitations of this study. First, the cut-off value of LMR applied in the enrolled studies was not uniform. This might have made a significant contribution to the substantial heterogeneity. Second, all of the included studies were retrospective. Third, excessive heterogeneity existed among the included studies. However, subgroup analyses showed that the heterogeneity diminished or disappeared in Caucasian patients and patients receiving surgery.

## 6. Conclusions

Our meta-analysis confirmed that low pretreatment LMR was associated with shorter OS, RFS, CSS, and worse clinicopathological features in patients with bladder cancer. Therefore, LMR could serve as a promising prognostic factor of bladder cancer.

## Figures and Tables

**Table 1:** Characteristics of the studies included in the meta-analysis.

Author	Year	Country	Ethnicity	Follow-up (months)	Treatment	No. of patients	Stage	Cut-off value	Survival analysis	Analysis	Confounding factors adjusted for	NOS score
Temraz	2014	Lebanon	Caucasian	24	Mixed	68	Mixed	2.81	OS/RFS	UV	NA	8
Lee	2015	UK	Caucasian	NA	Surgery	226	Early	1.8	OS	MV	Age, grade, tumor size, NLR, PLR	7
Zhang	2015	China	Asian	50.8	Mixed	124	Mixed	4	OS	MV	Age, sex, BMI, AC, concomitant Cis, T stage, LNM, DM, PLR	8
Yoshida	2015	Japan	Asian	72 (27.6-111.6)	Mixed	181	Mixed	3.51	OS	MV	T stage, N stage, grade, LVI, margin, AC, LVI	7
Lucca	2016	Austria	Caucasian	NA	Surgery	310	Early	3.3	OS	MV	T stage, grade, concomitant Cis, LVI, NLR, PLR, GPS, PNI	6
D'Andrea	2017	Austria	Caucasian	42.4 (18.3-85.1)	Surgery	4198	Mixed	3.5	OS/RFS/CSS	MV	Gender, age, margin, concomitant Cis, LNM, LVI, AC, NLR	8
Miyake	2017	Japan	Asian	22 (10–64)	Mixed	117	Mixed	3.3	OS/CSS	UV	NA	6
Rajwa	2018	Poland	Caucasian	14 (7-40)	Surgery	144	Mixed	2.44	OS/CSS	MV	T stage, LNM, grade, tumor necrosis, NLR, PLR	8
Wang	2018	China	Asian	NA	Mixed	270	Early	4	RFS	UV	NA	7

Abbreviations: OS: overall survival; RFS: recurrence-free survival; CSS: cancer-specific survival; MV: multivariate; Cis: carcinoma in situ; AC: adjuvant chemotherapy; NLR: neutrophil-lymphocyte ratio; PLR: platelet-lymphocyte ratio; LNM: lymph node metastasis; DM: distant metastasis; LVI: lymphovascular invasion; GPS: Glasgow prognostic score; PNI: prognostic nutritional index; PMI: psoas muscle index; NA: not available.

**Table 2:** Pooled hazard ratios (HRs) for OS according to subgroup analyses.

Subgroup	No. of studies	No. of patients	HR (95% CI)	*P* value	Heterogeneity	
					*I* ^2^ (%)	*P*_*h*_
Overall	8	5,368	0.63 (0.50-0.80)	<0.001	65.9	0.005
Ethnicity						
Asian	2	422	0.46 (0.25-0.87)	0.016	73.3	0.023
Caucasian	5	4,946	0.80 (0.71-0.89)	<0.001	8.2	0.360
Disease stage						
Early	2	536	0.61 (0.20-1.82)	0.377	0	0.705
Mixed	6	4,832	0.63 (0.49-0.80)	<0.001	75.3	0.001
Treatment						
Surgery	4	4,878	0.81 (0.74-0.89)	<0.001	0	0.883
Mixed	4	482	0.45 (0.27-0.73)	0.001	62.3	0.047
Cut-off for LMR						
≥3	5	4,930	0.56 (0.35-0.88)	0.011	76.6	0.002
<3	3	438	0.65 (0.41-1.04)	0.075	41.7	0.180
Analysis method						
Univariate	2	185	0.46 (0.27-0.79)	0.005	0	0.488
Multivariate	6	5,183	0.67 (0.53-0.86)	0.001	65.9	0.005

**Table 3:** Meta-analysis of the association between LMR and clinicopathological features of bladder cancer.

Characteristics	No. of studies	No. of patients	OR (95% CI)	*P*	Heterogeneity	
					*I* ^2^ (%)	*P*_*h*_
Age (≥60 vs. <60)	3	626	2.07 (1.22-3.50)	0.007	42	0.18
Gender (male vs. female)	4	4,818	1.18 (0.68-2.04)	0.56	70	0.02
Smoking status (always/current vs. never)	2	394	0.95 (0.63-1.45)	0.82	0	0.80
Differentiation (low vs. moderate/high)	5	4,886	1.60 (1.10-2.32)	0.01	35	0.19
Tumor size (>3 cm vs. <3 cm)	2	496	1.86 (0.74-4.71)	0.19	71	0.06
T stage (III-IV vs. I-II)	3	4,390	1.13 (1.01-1.28)	0.04	0	0.79
Lymph node metastasis (yes vs. no)	3	4,390	1.22 (1.06-1.39)	0.005	0	0.67
Distant metastasis (yes vs. no)	1	124	1.46 (0.37-5.73)	0.59	—	—
Multiplicity (multiple vs. solitary)	2	496	1.04 (0.68-1.58)	0.86	0	0.49
Concomitant Cis (yes vs. no)	2	4,322	0.88 (0.78-0.99)	0.03	0	0.87

Cis: carcinoma in situ.

## Supplementary Materials

Supplementary Materials The search strategies of this meta-analysis.
