# Supplementary material for: Prognostic Significance of the Lymphocyte-to-Monocyte Ratio in Bladder Cancer Undergoing Radical Cystectomy: A Meta-Analysis of 5638 Individuals
Source: Dis Markers. 2019 Apr 4;2019:7593560. doi: 10.1155/2019/7593560 (PMC6476040; doi:10.1155/2019/7593560)
Supplement: Supplementary Materials — The search strategies of this meta-analysis. [file 7593560.f1.doc]

**Search strategies：**

1. **CENTRAL:**

#1 MeSH descriptor Urinary Bladder Neoplasms explode all trees

#2 (bladder* NEAR/3 (cancer* or carcinoma* or neoplas* or tumo?r*)):ti,ab,kw

#3 MeSH descriptor Carcinoma, Transitional Cell explode all trees

#4 (tcc or transitional cell):ti,ab,kw

#5 MeSH descriptor Ureteral Neoplasms explode all trees

#6 MeSH descriptor Urethral Neoplasms explode all trees

#7 ((bladder* or urethra* or ureter* or urin* or urotheli* or renal pelvis or calice*) NEAR/3 (cancer* or carcinoma* or adenoma* or

adenocarcinoma* or squamous* or neoplas* or tum?r* or malignan*)):ti,ab,kw

#8 (#1 OR #2 OR #3 OR #4 OR #5 OR #6 OR #7)

#9 (LMR or lymphocyte to monocyte ratio or lymphocyte monocyte ratio or lymphocyte-to-monocyte ratio or lymphocyte-monocyte ratio).tw.

#10 #8 and #9

**2. MEDLINE:1946-**

1. exp urinary bladder neoplasms/

2. (bladder$ adj3 (cancer$ or carcinoma$ or neoplas$ or tumo?r$)).mp.

3. exp carcinoma, transitional cell/

4. (tcc or transitional cell).mp.

5. exp ureteral neoplasms/

6. bladder neoplasms/

7. urethral neoplasms/

8. ((bladder$ or urethra$ or ureter$ or urin$ or urotheli$ or renal pelvis or calice$) adj3 (cancer$ or carcinoma$ or adenoma$ or adenocarcinoma$ or squamous$ or neoplas$ or tum?r$ or malignan$)).tw.

9. or/1-8

10. (LMR or lymphocyte to monocyte ratio or lymphocyte monocyte ratio or lymphocyte-to-monocyte ratio or lymphocyte-monocyte ratio).mp.

11. 9 and 10

**3. Embase: 1974-**

1. exp bladder tumor/

2. (bladder$ adj3 (cancer$ or carcinoma$ or neoplas$ or tumo?r$)).mp.

3. exp transitional cell carcinoma/

4. (tcc or transitional cell).mp.

5. exp ureter tumor/

6. exp urethra tumor/

7. ((bladder$ or urethra$ or ureter$ or urin$ or urotheli$ or renal pelvis or calice$) adj3 (cancer$ or carcinoma$ or adenoma$ or

adenocarcinoma$ or squamous$ or neoplas$ or tum?r$ or malignan$)).tw.

8. or/1-7

9. (LMR or lymphocyte to monocyte ratio or lymphocyte monocyte ratio or lymphocyte-to-monocyte ratio or lymphocyte-monocyte ratio).mp.

10. 8 and 9
